# The molecular mechanisms and therapeutic strategies of EMT in tumor progression and metastasis

**DOI:** 10.1186/s13045-022-01347-8

**Published:** 2022-09-08

**Authors:** Yuhe Huang, Weiqi Hong, Xiawei Wei

**Affiliations:** Laboratory of Aging Research and Cancer Drug Target, State Key Laboratory of Biotherapy, National Clinical Research Center for Geriatrics, West China Hospital, Sichuan University, Chengdu, China

**Keywords:** Epithelial–mesenchymal transition, Metastasis, Tumor stemness, Circulating tumor cells

## Abstract

Epithelial–mesenchymal transition (EMT) is an essential process in normal embryonic development and tissue regeneration. However, aberrant reactivation of EMT is associated with malignant properties of tumor cells during cancer progression and metastasis, including promoted migration and invasiveness, increased tumor stemness, and enhanced resistance to chemotherapy and immunotherapy. EMT is tightly regulated by a complex network which is orchestrated with several intrinsic and extrinsic factors, including multiple transcription factors, post-translational control, epigenetic modifications, and noncoding RNA-mediated regulation. In this review, we described the molecular mechanisms, signaling pathways, and the stages of tumorigenesis involved in the EMT process and discussed the dynamic non-binary process of EMT and its role in tumor metastasis. Finally, we summarized the challenges of chemotherapy and immunotherapy in EMT and proposed strategies for tumor therapy targeting EMT.

## Introduction

The epithelial–mesenchymal transition (EMT) is a reversible process in which epithelial cells lose their properties and become mesenchymal cells, with altered expression of cell adhesion molecules and cytoskeleton. As a result, the cells develop motility-invasive properties, allowing them to transition between epithelial and mesenchymal states in a highly dynamic and plastic manner. The reverse process, which is known as mesenchymal–epithelial transformation (MET), occurs frequently during development (such as heart development, kidney morphogenesis, and somite formation) and cancer [1]. In living organisms, EMT is involved in embryogenesis, inflammation, fibrosis, wound healing, cancer development, and other physiological and pathological processes [2–5]. EMT is a continuous process of transition along the EMT spectrum, through which cells undergo loss of apical polarity, increase in anterior–posterior polarity, decrease in cell adhesion, shift from an epithelial to a mesenchymal phenotype, and gain of mesenchymal properties [6]. Biological processes of EMT, which can be classified as EMT-type 1, 2, and 3, are associated with embryogenesis, tissue regeneration, and cancer progression, respectively [7]. During embryonic development, EMT promotes pro-intestinal formation, neural crest stratification, mesodermal development, endocardial morphogenesis, and the generation of new cell and tissue types [6]. In wound healing and inflammation sites, EMT plays a central role in restoring epithelial and endothelial integrity. However, reactivation of EMT during pathological processes has an important role in cancer progression, as EMT can confer metastatic properties to tumor cells, enhance invasion, invade surrounding tissues, and colonize distant organs [8]. From the perspective of cancer cell dynamics, EMT is a dynamic and continuous spectrum process along the transition from the epithelial to the mesenchymal cell state. Tumor cells in the intermediate state of the epithelial to mesenchymal spectrum have both epithelial and mesenchymal properties and can better survive, metastasize and colonize distal organs [9].

The progression of EMT is regulated by the expression of EMT-translational factors (TFs) (such as SNAIL, ZEB, TWIST, and others) and miRNAs, as well as epigenetic and posttranslational regulators [10]. The three TFs of the SNAIL family (SNAIL1, SNAIL2, and SNAIL3) and those of the basic helix–loop–helix (BHLH) family (TWIST1 and TWIST2) can downregulate the expression of epithelial genes and upregulate the expression of mesenchymal genes. The ZEB family of zinc finger TFs (ZEB1 and ZEB2) can activate or repress transcription by binding E-box regulatory gene sequences. Noncoding miRNAs can also selectively bind mRNA to promote its degradation or inhibit its translation. Because a variety of miRNAs can act directly on the SNAIL family to regulate EMT, changes in miRNA expression affect the course and metastasis of EMT. Multiple signaling pathways such as TGF-β, Wnt, Notch, and PI3K-AKT are also involved in the regulatory network of EMT. Furthermore, post-translational regulation can induce EMT and promote metastasis of tumor cells. Epigenetic modifications and regulation can control the expression of related EMT-TFs, which are critical in regulating the molecular pathways of metabolism, transcription, differentiation, and apoptosis in the EMT process.

During cancer progression, cancer cells in EMT states are highly plastic when they transition to epithelial/mesenchymal states (i.e., partial EMT). Such a process is similar to cells during embryonic development, making them the drivers of tumorigenesis [11, 12]. Epithelial cells acquire some distinct mesenchymal features during cancer development that can be isolated in the primary tumor, enabling them to invade adjacent tissues before spreading distally. The phenotypic status of tumor cells undergoing this process can be graded by a combination of epithelial and mesenchymal markers. Individual cells that progress to different states along the E to M spectrum can generate extensive phenotypic heterogeneity within the tumor, and this phenotypic plasticity and heterogeneity can provide cancer cells with greater adaptability and resistance [13]. This is confirmed by the observation that skin and breast primary tumors have multiple E/M cell subpopulations with distinct chromatin landscapes and gene expression profiles, which are spatially localized to specific sites in the tumor [14]. Currently, several clinical trial studies applied novel personalized therapies based on molecular levels, which directly or indirectly inhibit EMT, using the expression of specific EMT markers as the selection criterion. Examples include the effect of aspirin on CTC subtypes (epithelial/mesenchymal/mixed) in metastatic breast and colorectal cancers [15]. Activation of EMT can facilitate the progression of this phenotype toward increased invasiveness and inhibit the sensitivity of tumor cells to chemotherapy by altering the microenvironment, where quasi-mesenchymal cells exhibit higher resistance to therapeutic regimens such as chemotherapy and immunotherapy [16, 17]. Notably, both EMT and MET are required for the process of metastasis. While EMT mobilizes the cells in the primary tumor, MET terminates the migration process and thereby resulting in the distal colonizing of cancer cells [6, 18, 19]. Furthermore, EMT is aberrantly activated during organ fibrosis and is required for the fibrotic response [17, 20]. EMT induces the formation of mesenchymal myofibroblasts near epithelial cells, which then aggregate and secrete fibrosis-promoting factors that promote tissue degeneration. Tissue regeneration is associated with organ fibrosis and disease progression and ultimately causes organ failure [21].

In this review article, we described the cytoskeletal and compositional changes that occur during the EMT process. The regulatory network of numerous regulatory factors in the EMT process was summarized, and the controversial topic of whether EMT is required for cancer development was discussed. The significance of partial EMT in promoting tumor cell plasticity and metastasis was also discussed. Furthermore, the role of EMT in cancer stem cells (CSCs) and circulating tumor cells (CTCs) was described. Finally, a summary of current EMT therapeutic modalities was presented, as well as the various salient issues and challenges associated with the current therapeutic processes.

## Cytoskeletal changes between epithelial and mesenchymal cells

**Fig. 1:**
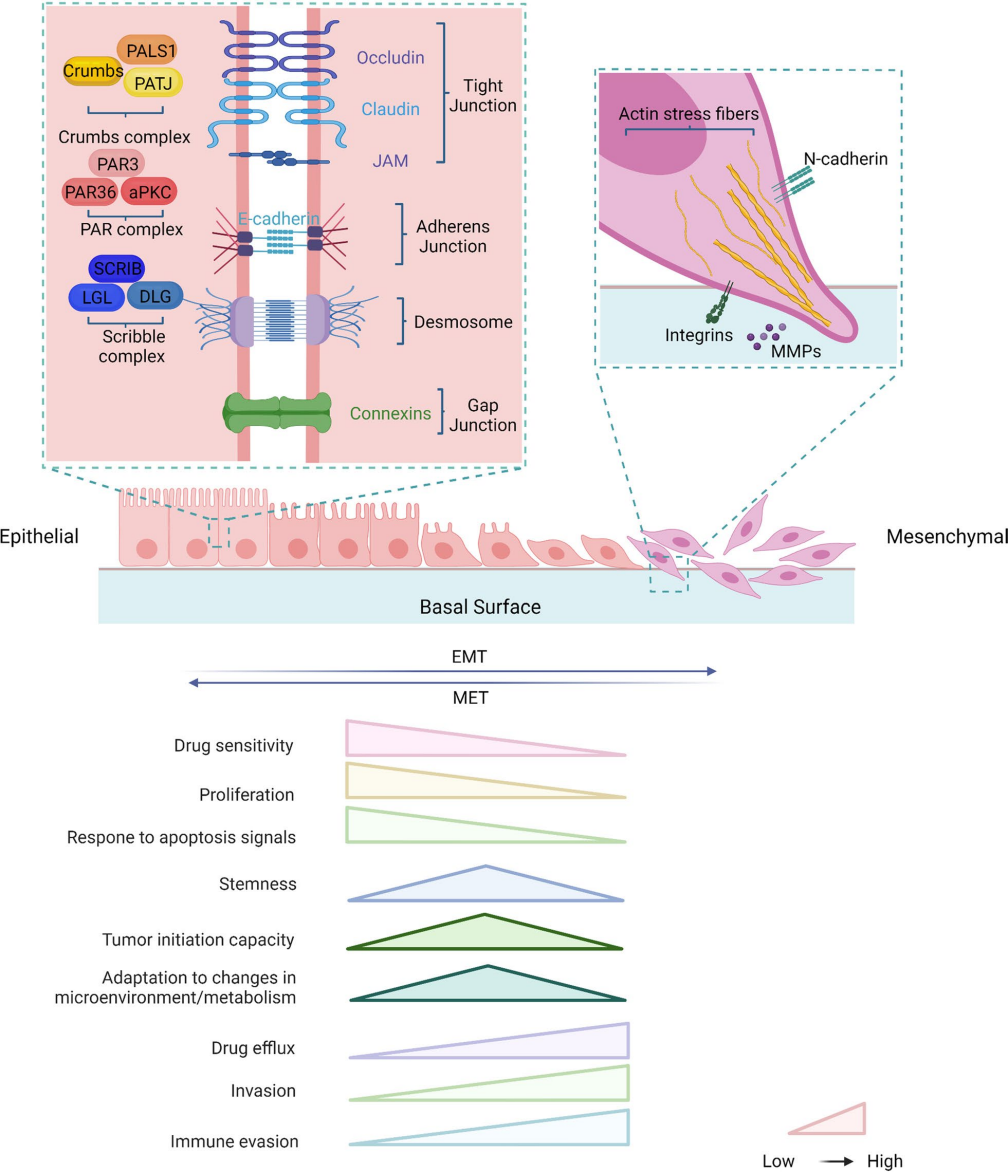
Changes in the cytoskeleton and properties between epithelial and mesenchymal during the EMT process. Epithelial cells exhibit apical–basal polarity with cell–cell and cell–matrix attachment. Three multi-protein complexes (Scribble complex, Crumbs complex, and PAR complex) interact to regulate the spatial separation of apical and basal structural domains together to establish cell polarity. Intercellular adhesion and communication are provided by intercellular junctions and maintain tissue stability and integrity. Tight junctions (TJs) form strips around cells that help separate apical and basal regions and form sealed spaces between adjacent cells, preventing the flow of material. Adherens junctions (AJs) are located below TJs, surround cells, and provide intercellular adhesion, but they are relatively permeable. Gap junctions are gaps located on the outer surface of cells and are hydrophilic ion transport channels between adjacent cells. Bridging granules provide sites of cell adhesion and intermediate filament binding to disklike structures located on the outer surface of the cell. The occurrence of EMT leads to the dissolution of intercellular junctions and loss of cell polarity allowing cytoskeletal rearrangements that alter the shape of the cell, transforming the cell into a mesenchymal phenotype and promoting cell motility and invasion. Based on a synthesis of the literature, we conclude that as the EMT progresses, the cell characteristics are changed, including reduces in drug sensitivity, proliferation, and response to apoptosis signals and increases in drug efflux, invasion, and immune evasion. The partial EMT with intermediate state has properties of enhanced stemness and tumor initiation capacity, and stronger ability to adapt to the changes in immune microenvironment and metabolism

Cells differentiate into different states throughout the development of an organism, and they can be broadly classified into epithelial or mesenchymal phenotypes [7, 22]. Epithelial cells have a flat and polygonal shape that is maintained synergistically by the actin cytoskeleton and intermediate filaments [23]. Epithelial cells have a strong apical and basal polarity, with plasma membranes oriented toward and away from the lumen, respectively [24]. Each membrane is composed of different proteins that allow for the targeted transport of molecules and the localization of various activities to specific cellular regions [25]. Therefore, apical and basal polarization is imperative for many biological functions such as endocytosis, exocytosis, and vesicle transport [26]. Additionally, adjacent individual epithelial cells develop tight junctions and coalesce into a single cell layer to form the basic epithelial tissue [27, 28]. Epithelial cell mobility is limited by shape and attachment, allowing them to migrate only in patches and whole blocks [29]. Epithelial cells usually communicate with each other through tight junctions, adherence junctions, desmosomes, and gap junctions [30] (Fig. 1). When epithelial junctions are dissolutive, epithelial cells lose their apical–basal polarity [30].

Mesenchymal cells are distinguished from epithelial cells by their irregular morphology, with an elongated, spindle-shaped form and less rigid topography [26]. They are also characterized chiefly by the acquisition of anterior–posterior polarity while losing cellular adhesion or uniform composition. In mesenchymal cells, keratin is downregulated, whereas vimentins are upregulated, which increases the strength of the cytoskeleton and flexibility, and the ability to migrate and invade tissues [31]. In parallel, bundles of stress fibers are structured by actin filaments to generate new actin-rich membrane protrusions, allowing for various types of movement and sensory reception [32].

EMT is also a process that routinely promotes the migration of cells toward the extracellular matrix, which is known as invasion. The process of invasion differs from the controlled and seemingly stable interactions between epithelial and endothelial cells in intact tissue, as well as from the underlying basement membrane [33]. To undergo directed cell migration, as shown for high-grade cancer cells, epithelial cells must divert their apical–basal cell polarity to frontal polarity [20]. In vertebrate cells, polarization is usually regulated by the apical compartment (partitioning‑defective (PAR) and Crumbs complexes) and the basolateral compartment (Scribble complexes), and disruption of these complexes confers loss of apical–basal polarization during EMT [20, 34]. The Scribble complex is considered a tumor suppressor that is found in the basal structural domain, which maintains basolateral polarity [35]. The PAR complex defines the boundary between the basal and apical structural domains, while the Crumb complex is associated with the LIN-1 (PALS1) related protein (PALS1) and the PALS1-associated tight junction (PATJ) complex, which controls apical structural domain formation [20, 36]. These two complexes are located in tight junctions and work together to maintain the apical structural domain.

The PAR and Scribble complexes are antagonistic to each other, and the PAR complex helps in enhancing the activity of the Crumb complex. Apical–basal polarity is associated with the integrity of epithelial or endothelial junctions, whereas redirected cell polarity is associated with the deconstruction of lateral cell–cell junctions of the epithelium or endothelium during EMT [20]. Tight junction instability is accompanied by decreased expression of claudin and occludin, and the diffusion of zonula occludens 1 (ZO1) from cell contacts [20, 30]. During transformation, the expression or function of epithelial genes such as E-cadherin-specific cytokeratin and zone of occlusion 1 (ZO-1) is lost, whereas the expression of genes defining the mesenchymal phenotype (such as vimentin, fibronectin, N-cadherin, β1 and β3 integrins) is enhanced [18, 37, 38]. Initiation of EMT also disrupts bridging granules, allowing low levels of connexin to compromise the integrity of gap junctions [39]. When adhesion junctions are destabilized, epithelial calmodulin (E-cadherin) on the cell membrane is cleaved and degraded [29]. Changes in the aforementioned molecular readouts can characterize the specific cellular features of the process [40].

Epithelial–mesenchymal transition cells can develop a spindle-shaped mesenchymal morphology and motility by forming protrusions due to the reorganization of the actin cytoskeletal structure and the aforementioned changes [14]. The cortical organization of the actin cytoskeleton is repositioned as bundles of stress fibers and clustered near the ventral surface of cultured cells [24]. The stress fiber bundles facilitate various types of movement and sensory reception by forming new actin-rich membrane protrusions [31]. Furthermore, keratin downregulation and vimentins upregulation strengthen the cytoskeleton, making it less susceptible to damage during migration and more flexible. In addition, cells with EMT characteristics can degrade and invade their extracellular matrix at the invasion front of individual cells or populations of cells by activating proteases such as matrix metalloproteinase (MMPs) [41].

In addition, tumor cells in epithelial–mesenchymal hybrid state or partial EMT during the transition have the greater migratory ability, allowing them to detach from their original tissue and wander throughout the body [14]. Besides, changes in the intracellular and intravascular environment make EMT complex and diverse [24]. According to ongoing research, EMT represents a spectrum of states with intrinsic plasticity, progressively forming different intermediates for transformation rather than being determined by only two cellular states [6]. Certain EMT states exhibit both epithelial and mesenchymal cell characteristics in both in vitro and in vivo models of many developmental and disease processes [14]. These EMT cells may co-express epithelial and mesenchymal markers or may lose epithelial markers without obtaining mesenchymal markers [42]. Therefore, the cellular and molecular characteristics of EMT should be evaluated in a context-dependent manner. We described “partial EMT” in greater detail in the subsequent section.

## Regulatory networks of EMT

### Transcriptional regulation of EMT

**Fig. 2:**
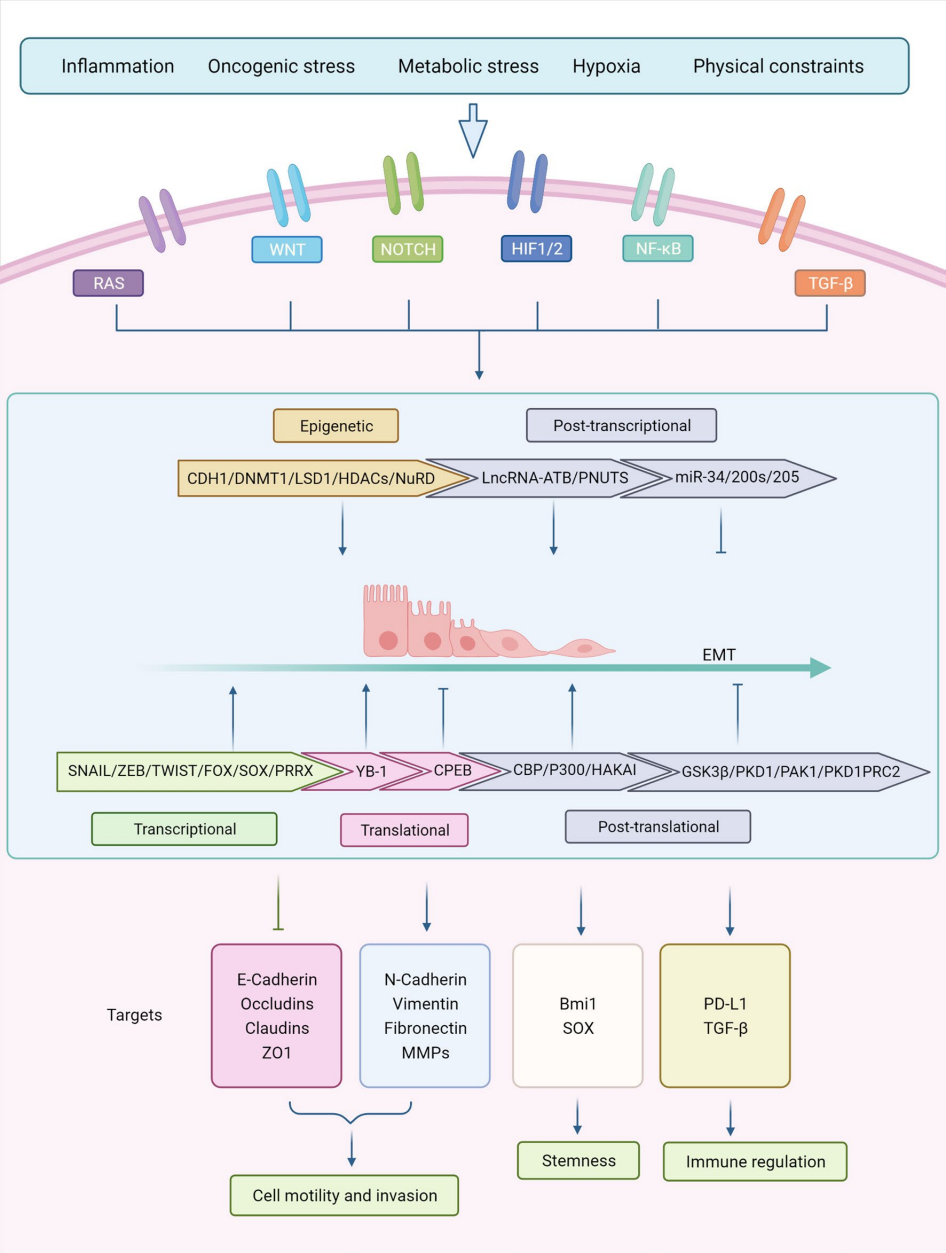
Regulatory network of EMT. EMT is tightly regulated by a complex network which is composed of several factors, including transcription regulation, posttranslational control, epigenetic modifications, and noncoding RNA-mediated regulation. Snail, Twist, Zeb, and other EMT-related transcription factors are regulated by multiple signaling pathways at post-transcriptional and posttranslational level and orchestrate with other epigenetic factors to regulate downstream transcriptional networks, further mediating the biological effects of EMT

The transition of cells from epithelial to mesenchymal states is mediated by key transcription factors, which primarily regulate intercellular adhesion, cell polarity, and motility [18]. Transcription factors induce mesenchymal gene expression by suppressing genes associated with the epithelial phenotype, resulting in the EMT cellular signature [43]. The major EMT-inducible transcription factors are Zinc finger binding transcription factors SNAIL1 and SNAIL2, zinc finger E box binding homology frame factors ZEB1 and ZEB2, and basic helix–loop–helix (BHLH) factors TWIST1 and TWIST2 [20]. SNAIL1/2, ZEB1/2, and TWIST1/2 are thought to be major regulators of the transcriptional pathway that drives EMT, and they converge to activate the expression of transcription factors (Fig. 2) [44].

SNAIL1 and SNAIL2 have critical roles in the activation of the EMT program during embryonic development, fibrosis, and cancer [45]. In a physiological context, they enhance neural crest development and migration [46]. SNAIL1 and SNAIL2 bind to the E box sequence of the CDH1 promoter region and directly repress transcription by recruiting the polycomb repressor complex (PRC), which contains components such as G9a, methyltransferases enhancer of zeste homolog 2 (EZH2), histone deacetylases 1, 2, and the Lys‑specific demethylase 1 (LSD1) [47]. All of these factors coordinate histone hypermethylation and deacetylation to repress epithelial gene expression [47–50]. SNAIL1 could directly inhibit TJ formation and epithelial markers such as E-cadherin and claudins while upregulating mesenchymal phenotypic markers including vimentin and fibronectin [51–53]. SNAIL2 promotes the loss of cell adhesion and polarity and confers migration and invasion capabilities [54]. Moreover, they both cooperate with other transcription regulators to control gene expression. For example, SNAIL1 cooperates with ETS1 to activate MMP expression [55]. SNAIL expression in specific physiological settings could be activated via multiple signaling pathways, including TGF-β, Wnt, Notch, and growth factors acting on receptor tyrosine kinases (RTKs) [56], indicating that the SNAIL-associated EMT program is driven by multiple mechanisms. SNAIL1 and SNAIL2 play key roles in EMT-induced tumor progression. For example, the expression levels of SNAIL1 could be used as a prognostic indicator. E-cadherin expression is positively correlated with patient survival, whereas overexpression of MMPs is associated with tumor cell aggressiveness [57]. Furthermore, it has been shown that stable SNAIL2 expression decreases e-calmodulin levels and thus enhances metastasis in breast and ovarian cancers [58, 59].

ZEB1 and ZEB2, which belong to the human ZEB family, are zinc finger TFs that bind to regulatory gene sequences at E-box and activate or repress transcription [60]. ZEB-mediated transcriptional repression usually involves the recruitment of C-terminal-binding protein (CTBP) [61, 62]. In certain cancer cells, ZEB interacts with the switch/sucrose nonfermentable (SWI/SNF) chromatin-remodeling protein BRG1 (Brahma-related gene 1) to suppress gene transcription [63]. Furthermore, ZEB1 can switch from a transcriptional suppressor to an activator by interacting with co-activators PCAF and P300 [64]. ZEB proteins suppress the expression of cell polarity complex components and downregulate TJ genes, thereby driving EMT [65]. However, ZEB proteins enhance the expression of the mesenchymal protein vimentin and N-cadherin [66]. The TGF-β and Wnt signaling pathways, as well as other growth factors that activate the Ras-MAPK signaling pathway can all induce ZEB protein expression [20]. TWIST1 can cooperate with SNAIL1 to induce ZEB1 expression, which is often followed by SNAIL activation [67]. According to genetic evidence from related studies, ZEB1 expression is essential for effective invasion and metastasis in mouse models of pancreatic cancer [68]. The expression of ZEB1/2 in epithelial cells causes an EMT and mesenchymal phenotype in tumor stem cells, which promotes invasion, metastatic dissemination, and a dedifferentiated state [69]. Moreover, ZEB1 expression is associated with poor clinical outcomes in solid tumors (including breast, colorectal, and pancreatic tumors) [70–73], and poor prognosis and survival in various tumor types [74]. However, it is worth noting that ZEB1 and ZEB2 have opposite effects on different cell lines, implying that tissue context is critical for the function of EMT-TF [64, 75, 76]. For instance, in a mouse model of melanoma, ZEB2 inhibited tumor metastasis, while ZEB1 drove tumorigenesis and progression. In clinical studies, ZEB2 deficiency was associated with reduced survival in melanoma patients, while ZEB1 expression was associated with poorer clinical outcomes. Additionally, ZEB1 and ZEB2 can have different functions due to the regulation of the transforming growth factor-β (TGF-β) and bone morphogenetic protein (BMP) signaling pathways. ZEB1 induces osteoblast differentiation and associated growth arrest, whereas ZEB2 has opposite functions.

TWIST1 and TWIST2 belong to the basic helix–loop–helix (BHLH) family of transcription factors. Both proteins form homodimers or heterodimers with E12 and E47 to regulate E-box DNA response elements in order to suppress or activate transcription [77]. TWIST1, similar to SNIAL, can suppress the expression of E‑cadherin and promote the expression of N‑cadherin, resulting in decreased cell adhesion and increased cell motility [78, 79]. The transcriptional regulation of TWIST proteins is essential for the recruitment of methyltransferase SET8 or activation of B lymphoma Mo‑MLV insertion region 1 homolog (BMI1) [80, 81]. A related study found that SNAIL2 knockdown blocked the ability of TWIST to activate EMT in mammary cells, suggesting that TWIST can indirectly induce transcriptional repression of E- cadherin. [82]. Multiple signaling pathways activate TWIST during the EMT program. Importantly, the hypoxia-inducible factor-1α (HIF-1α) transcription factor activates TWIST expression and promotes EMT and tumor cell dissemination under hypoxic conditions [78]. Related studies, using a mouse model of spontaneous squamous cell carcinoma, demonstrated that cancer cells undergo EMT and spread to the circulation, which is facilitated by activation of the EMT-inducible transcription factor TWIST1 [79]. Furthermore, TWIST has been shown to play a crucial role in the development of benign skin tumors in mice. Conditional ablation of TWIST expression in the skin prevented DMBA/TPA-induced skin cancer and significantly decreased tumorigenesis [83]. Moreover, TWIST overexpression is associated with tumor invasion and metastasis [84, 85].

In addition to SNAIL, ZEB, and TWIST, the EMT program is regulated by various transcription factors in tissue development and cancer. For example, several forkhead box (FOX) proteins, such as FOXC1, FOXC2, and FOXQ1, can promote mesenchymal differentiation and decrease the expression of proteins involved in polarity complexes and cell–cell junctional [86–88]. SRY box (SOX) transcription factors cooperate with SNAIL proteins to promote EMT [89]. According to research, paired-related homeobox 1 (PRRX1) protein is a novel regulator of EMT [90]. However, the interaction between various EMT-TFs needs to be clarified. In addition, the precise regulation and functions of the EMT-TFs in different EMT contexts should be further investigated.

### Translational and post-translational control

Translational control has a significant effect on EMT (Fig. 2). The forced expression of Y-box-binding protein-1 (YB-1) induces EMT and promotes metastasis by directly activating the cap-independent translation of SNAIL1 mRNA and other mesenchymal factors in RAS-transformed mammary epithelial cells [91]. Embryonic lethal abnormal vision-like RNA promotes the EMT process by enhancing the stability of SNAIL1 mRNA [92]. In breast cancer, cytoplasmic polyadenylation element-binding protein 1 (CPEB1) mediates EMT and metastasis by enhancing the shortening of the polyA tail of MMP9, which lowers MMP9 translation [93].

Recent studies have underlined the importance of post-translational modification at the proteome level for the EMT program. Protein phosphorylation, the most common post-translational modification, is required for the regulation of multiple molecular pathways in metabolism, transcription, differentiation, and apoptosis [94]. As a post-translational modification, phosphorylation can control the expression of SNAIL [95]. Glycogen synthase kinase-3β (GSK-3β), a classical kinase involved in many signaling pathways, phosphorylates SNAIL through two consecutive motifs, thereby controlling its ubiquitination and subcellular localization. First, GSK-3β binds to and phosphorylates Ser97 and Ser101 in the SNAIL motif 1 to induce nuclear export of SNAIL. Subsequently, GSK-3β phosphorylates Ser108, Ser112, Ser116, and Ser120 in motif 2 to promote β-Trcp-mediated degradation of the SNAIL proteasome [96]. Several signaling pathways, including Wnt, NF-κB, Notch, and PI3K-AKT, inhibit GSK-3β‑mediated phosphorylation or disrupt the GSK-3β-SNAIL interaction to boost SNAIL stability and downregulate E-cadherin, resulting in EMT program activation [96–99]. Protein kinase D1 (PKD1) phosphorylates SNAIL1 and promotes its export from the nuclear [100]. On the contrary, small C-terminal domain phosphatase 1 (SCP1) could dephosphorylate SNAIL1 to retain it in the nucleus and enhance its activity [101]. Several other kinases, including p21-activated kinase 1 (PAK1) and large tumor suppressor 2 (LATS2), phosphorylate SNAILS, affect its activity positively or negatively [102, 103]. Furthermore, phosphorylation regulates the stability of TWIST. TWIST phosphorylation at Ser68 by MAPKs prevents ubiquitin-mediated degradation and enhances TWIST activity [104].

Other post-translational modifications can modulate the course of EMT by regulating the activity of key EMT-TFs. Sumoylation of ZEB2 by PRC2 promotes its export from the nucleus, abolishing ZEB-mediated gene regulation [105]. The stability and activity of EMT-TFs are regulated by ubiquitination, which activates or suppresses the EMT program. An atypical ubiquitin E3 ligase complex, Skp1-PamFbxo45, controls the EMT program by regulating the degradation of different EMT-TFs [106]. In breast cancer, ZEB1 is regulated by the E3 ubiquitin ligase SIAH, which marks its degradation [107]. However, deubiquitination of ZEB1 by the ubiquitin-specific protease-51 (USP51) promotes its stabilization [108]. The same post-translational regulation of epithelial proteins is possible during EMT. Hakai, an E3 ubiquitin ligase, ubiquitinates E-cadherin, inducing its endocytosis and destruction [109]. Acetylation of SNAIL1 protein by CBP inhibits the formation of the suppressor complex and converts SNAIL1 from a gene repressor to an activator [110]. Moreover, p300 can acetylate SNAIL1 and TWIST1, regulating their stability, location, and interactions with other proteins [111]. Members of the miR-200 family act as oncogenic miRNAs, enhancing E-cadherin expression while suppressing the expression of ZEB1 and ZEB2. The sumo modification of FoxM1 at lysine 463, which is a posttranslational modification, is required for complete suppression of miR-200b/c in breast cancer cells [112].

### Epigenetic modifications

Epithelial–mesenchymal transition is associated with important epigenetic alterations, which are often required to mediate the function of EMT-TFs [113] (Fig. 2). Over the past few decades, specific modifications, including DNA methylation and histone modifications, as well as multiple epigenetic regulators, have been identified as key regulators of the EMT process. In a variety of human tumors including breast, bladder, lung, liver, gastric, and prostate cancers, CDH1 promoter methylation has been implicated as a key factor in EMT [114–116]. Transcription factors such as SNAIL, SLUG, ZEB1, and ZEB2/SIP1 bind to the E-box on the CDH1 promoter and are considered to be direct inhibitors of E-cadherin [117–119]. Research indicates that ZEB1 mediates CDH1 downregulation in basal cell-like breast cancer and recruits DNMT1 to the CDH1 promoter to maintain the methylation status of the promoter [120]. The histone demethylase LSD1, also known as KDM1A and AOF2, plays an essential role in EMT [121]. It was discovered that SNAIL interacts with LSD1 through its SNAG (SNAIL1/GF) structural domain and recruits LSD1 to the CDH1 promoter. Therefore, the methyl group on lysine 4 of histone H3 will be removed (H3K4m2) [48]. Other demethylases such as KDM6B and PHF8, as well as the methyltransferases PRMT5, EZH2/SUZ12, SUV39H1, and G9a have been reported to regulate EMT by regulating the expression of EMT-TF (mainly SNAIL1 and ZEB) or interacting with these factors to affect the expression of downstream genes [122–126].

During the transition of trophoblast stem cells from an epithelial to a mesenchymal state, the histone deacetylase HDAC6 directly deacetylates the promoter of the TJ gene, resulting in decreased cell–cell adhesion, which is one of the earliest EMT events [127]. The ZEB and TWIST families of transcription factors also bind and recruit the nucleosome remodeling deacetylase NuRD complex to their target promoters [128]. Altogether, epigenetic modifications are the basis for determining the expression of key proteins in the EMT. Notably, these modifications are often reversible and can play a key role in defining EMT plasticity.

### Noncoding RNA-mediated regulation

Several microRNAs (miRNAs) can directly regulate the expression of EMT transcription factors (Fig. 2). Noncoding miRNAs inhibit the translation or promote degradation of mRNAs by selectively binding to them. The miR-200 family, which has five miRNAs, and miR-205 suppress ZEB1/2 expression [129, 130]. Although they target different ZEB sequences, they cooperate to enhance ZEB suppression [131]. In liver carcinoma cell, p53 inhibits the EMT program by increasing the levels of miR‑200 and miR‑192/miR-215, resulting in low expression of ZEB1/2 [132]. In colorectal, breast, prostate, and hepatocytes cancer cells, miR-34, miR-203, miR-29b, and miR-30 can similarly suppress the expression of SNAIL1 [133–136]. Furthermore, miR-1 and miR-200 can suppress the expression of SNAIL2 in prostate adenocarcinoma cells [137]. The expression of miRNAs and EMT-TFs are regulated in a double-negative feedback loop. It is widely known that the miR-200 family suppresses the production and activation of ZEB, which in turn suppresses the expression of miR-200 family [129]. In addition, similar to the activities of miR-200 family members, miR-1199-5p acts as a guardian of epithelial cell phenotype in a reciprocal double-negative feedback loop with ZEB1 [138]. Other double-negative feedback loops operate between miRNAs and EMT-TFs were reported in multiple processes of tumor epithelial–mesenchymal transition, including miR-34/miR-203 and SNAIL1, miR-1/miR-200 and SNAIL2, miR-33a-5p and ZEB1, miR-145 and ZEB2, miR-200 and Foxf2, miR-30a and SOX4, or miR-15a/16-1 and AP4 [133, 134, 137, 139–143]. These feedback loops may explain how imbalanced expression between microRNAs and EMT-TFs causes reinforced activation of EMT and steady mesenchymal specification once EMT is completed, as well as the reversibility of EMT and MET [144].

Furthermore, miRNAs also regulate the expression of EMT-TFs indirectly, thereby controlling EMT progression. miRNA let-7 and miR-365 control the expression of SNAIL1 and TWIST by deregulating high-mobility group A2 (HMGA2), a chromatin-binding protein that activates SNIAL and TWIST [145, 146]. In addition to regulating major transcription factors, miRNAs could regulate EMT by directly interacting with epithelial or mesenchymal genes [20]. For example, the miR-9 increased the motility and invasiveness of tumor cells by directly suppressing the expression of the E-cadherin-encoding messenger RNA [147]. In addition, miR-194 directly interacted with several 3′ untranslated regions (3′-UTR) of multiple mRNAs such as N-cadherin mRNA and lowers its expression in advanced-stage gastric cells [148]. Overexpression of miR-194 inhibited migration, invasion, and metastasis of hepatic cancer cells [149]. Furthermore, several miRNAs, including miR‑491‑5p, miR‑155, miR‑24, and miR‑124, regulate EMT progression by targeting cell architectural components [150–153].

Long noncoding RNAs (lncRNAs) are also involved in EMT regulation. For example, lncRNA-activated by TGF-β (lncRNA-ATB) and lncRNA-PNUTS are thought to act as sponges of the miR-200 family and miR-205, respectively, and isolation of these miRNAs prevents them from suppressing EMT-TF transcription [154, 155]. In addition, translational regulator lncRNA negatively regulates the translation of CDH1 mRNA to promote EMT [156]. H19 can mediate EMT by differentially binding to miR-200b, miR-200c, Let-7, SNAIL2, and Ezh2 [157]. In EMT cases of murine and human breast cancer, lncRNA EMT-associated transcript 20 (ET-20) was found to be transcribed in an antisense manner through the Sox4 EMT master transcription factor and co-regulates with the Tnc gene to bind to bridging proteins on the cell membrane. This process thus results in the impairment of intercellular junctions and enhancement of EMT. [158]. In conclusion, the regulatory activities of miRNAs and lncRNAs as post-transcriptional regulators form a complex regulatory network that controls EMT. In particular, while miRNAs control EMT by regulating EMT transcription factors or regulators, overexpression of lncRNAs in various cancers can induce EMT and promote tumor metastasis.

## The relationship between EMT and tumors

**Fig. 3:**
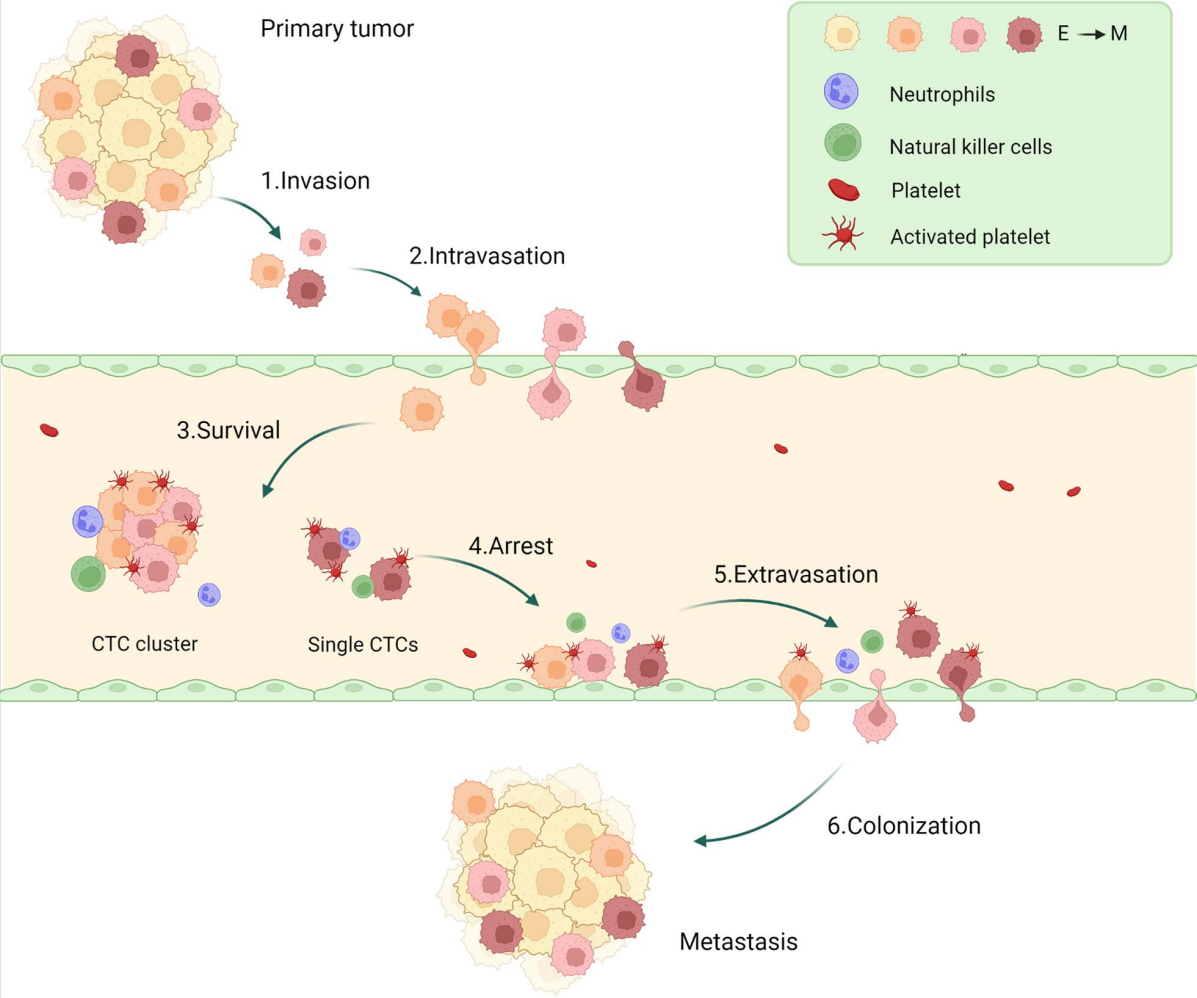
Role of EMT in the tumor metastasis. Tumor cells in situ are induced by EMT to initiate the metastatic cascade process. Intermediate state and mesenchymal stem cells (MCSCs) lose intercellular junctions, detach from tumor tissue, and invade surrounding tissues. Metastatic tumor cells enter the circulation through the endothelial barrier by active or passive trans-endothelial migration (TEM), invading the surrounding mesenchyme and disrupting endothelial junctions. After entering the circulation, single or clustered circulating tumor cells (CTCs) can bind to coagulation factors on platelets by expressing thrombin, forming a unique immune mechanism that protects metastatic cells from immune escape. Neutrophils are also recruited and bind to CTCs to promote tumor cells survival and proliferation. CTCs move slowly, roll along the endothelium, and then arrest. MCSCs anchor with endothelial cells for extravasation and then colonize distal organs via the MET process

Unlike normal tumor cells, cancer stem cells (CSCs) have the function of initiating and maintaining tumor growth, self-renewal, and proliferation. As a subgroup of CSCs, metastatic cancer stem cells (MCSCs) can receive matrix signals from the distal organ environment and escape from the boundary of the primary tumor [24]. Activation of EMT promotes the invasive phenotype of MCSCs, and makes MCSCs participate in the cascade process which is composed of three steps: first, the tumor cells invade to surrounding tissues; second, the trans-endothelial migration of tumor cells into the circulation system; third, the tumor cells colonize in distal tissue which results in the formation of metastatic foci (Fig. 3). EMT-TFs act as key regulators of CSC; thus, EMT is closely related to the acquisition of tumor cell stemness.

### The roles of EMT and MET in tumor metastasis

During the process of cell detachment from in situ, the downregulation or degradation of E-cadherin and the upregulation of N-cadherin destabilize AJs and disrupt the intercellular junctions of MCSCs, promoting cell migration and invasion [29, 159]. The activation of the “cadherin switch” can be used as a marker for the initiation of EMT. By regulating pro-apoptotic and anti-apoptotic genes, the “integrin switch” enables cells to make use of survival signals through overexpression or structural activation of integrins, and then changes the cell metabolism to prevent MCSCs from losing their nest due to apoptosis [160, 161]. During tumor cell invasion, the response to internal and external signals of the cell promotes the formation of stress fibers that inhibit cofilin and actin, allowing the cytoskeleton to reorganize dynamically [162]. The attachment sites formed by protrusions of actin between the cell and the ECM promote cell elongation and mesenchymal migration enabling cell motility [163]. The master cell guides cells that maintain intercellular junctions to migrate in narrow lines, clusters, or broadsheets, and the phenotype of the cells is influenced differently by the following cells [164, 165]. In contrast, cells that have lost their intercellular junctions can migrate in both mesenchymal and amoeboid forms [162].

After the early steps of local invasion of the metastatic tissue by EMT cells, MCSCs located at the tumor front begin to invade the surrounding tissues into the blood vessels or lymphatic vessels. EMT is a process in which cells transition from epithelium to mesenchyme and leading to the emergence of various hybrid phenotypes [13, 17, 41, 166]. The E/M hybrid phenotype, for example, is crucial in the metastatic spread of tumor cells because it has a high degree of epithelial–mesenchymal plasticity (EMP), a property that makes it heterogeneous during metastasis and contributes to better tumor cell aggregation and dissemination [167]. In related studies, a mixed E/M phenotype was found in heterogeneous circulating tumor cells (CTCs) in human lung and breast cancer patients [168–170]. During the intravasation process, MCSCs invade the surrounding stroma and disrupt endothelial junctions in order to transcend the endothelium barrier into the circulation by active or passive migration for trans-endothelial migration [171].

The expression of Notch, VEGF, and TGF-β signaling pathways have significant effects on endothelial function. Histone proteases can degrade BM and ECM components and activate urokinase-type plasminogen activator (uPA), which can then mediate ECM remodeling [172]. Matrix metalloproteinases can cleave E-cadherin, induce EMT via EGFR signaling, and promote neoangiogenesis by releasing growth factors from degraded ECM [173]. By expressing thrombin, MCSCs can bind to coagulation factors on platelets upon entry into the circulation, forming a unique immune mechanism to protect metastatic cells from immune escape and maintaining mesenchymal properties by activating the SMAD and Notch pathways [174, 175]. Collectively, migrating cells or aggregated individual cells in the circulation system can form swarms of mobile metastatic cells, which have better survival and metastatic potential [176, 177]. Next, the metastatic cells can find a suitable microenvironment to protect themselves from apoptosis and retain at the new distant metastasis sites. Then they migrate and exudate across endothelial cells and invade the tissue around the blood vessels. This homing mechanism and circulatory pathway enable MCSCs to migrate to and colonize distant organs [178].

The motility of circulating metastatic cells is slowed in capillaries that are similar in size to the cells themselves, which roll along the endothelium before arresting [179]. The binding of intercellular adhesion molecules (ICAM1), galactose lectin 3, and selectin expressed on endothelial cells to integrins, CD44, and MUC1 expressed on metastatic cells mediates the anchoring of MCSCs to the endothelium. Tumor cells eventually colonize through the MET process, and metastatic cells reacquire epithelial features during EMT reversal, generating distal secondary tumors that are histopathologically similar to the primary tumor [171]. A study examined mammary tumor cells in mice using in vivo microscopy techniques and found that they can undergo the EMT process spontaneously and revert to an epithelial state after mobility, migration, invasion, and colonization, corroborating the aforementioned process [180].

### EMT is the key driver of tumor metastasis

EMT is a multidimensional and nonlinear process, it is difficult for in vitro experiments to accurately reflect the dynamic EMT process that cancer cells undergo in vivo [181]. Consequently, most of the available studies are based on cultured cell lines or xenograft models. In vivo studies focus on clarifying the characteristics of EMT by injecting or xenografting parental or manipulated cancer cells, whereas in vitro experiments mainly investigate the functional role of EMT-TFs through their acquisition or loss. EMT is hypothesized to be a driver of cancer progression. In related studies, researchers traced the process of SNAIL1 endogenous expression in tumors and found SNAIL1 activation and EMT in primary tumor cells, which eventually spread [182]. Furthermore, a decrease in SNAIL was found to inhibit the development of metastasis in the PyMT breast cancer model [183]. In another breast cancer model, the absence of TWIST resulted in a decrease in tumor metastasis [184]. Deletion of ZEB1 prevents tumor cells from invading and migrating [68]. In melanoma patients, ZEB2 deletion reduced their survival rate, which was verified in a mouse model of the disease [75, 76]. Therefore, these studies suggest that EMT is important for tumor metastasis.

### Context-dependent EMT program

Although the EMT-TFs are the key drivers of tumor initiation, progression, and metastasis, several controversial results and conflicting data about the crucial role of EMT and EMT-TFs in cancer metastasis continue to be extensively debated [185–189]. It was observed that deletion of SNAIL or TWIST did not significantly inhibit metastasis of tumor cells in genetically engineered mouse models of pancreatic ductal adenocarcinoma [186]. In addition, a study conducted by Fisher et al. developed and used a mesenchymal-specific Cre-mediated fluorescent labeling switch system to track EMT in spontaneous breast-to-lung metastasis and found that a small proportion of primary epithelial tumor cells manifested EMT phenotype, but lung metastasis was achieved using non-EMT cells [185]. In a relevant mouse breast cancer model, by tracking the endogenous SNAIL activation, it was found that only primary tumors had SNAIL1 activation and endogenous EMT production [182, 190]. In the MMTV-PyMT mouse model of metastatic breast cancer, E-cadherin gene-negative, as well as N-cadherin gene-positive tumor cells undergoing EMT, were found to be the cells that migrated and possibly initiated the metastatic cascade. But vimentin gene activity was not detected during metastasis. These studies confirmed the presence of EMT but did not demonstrate its necessity for metastasis, suggesting that tumor cells may use the unique EMT gene program to metastasize and colonize distant organs [180, 191]. Therefore, the unnecessary association between EMT and metastasis may be influenced by context-dependent, which can be varied in different tumor types with different EMT requirements.

An excellent review has recently discussed the non-redundant functions of each particular EMT-TF and demonstrated that the tissue context is critical for the precise functions of EMT-TFs due to their distinguishing expression in development, tissue homeostasis, and different tumor types [43]. Even with the similar morphology of migratory tumor cells, carcinoma cells that have undergone EMT program in different tumor types may be regulated by diverse gene expression profiles [42]. A good example is that the CTCs from patients with lobular breast cancer display epithelial phenotypes, whereas those from HER2+ and triple-negative subtypes exhibit mesenchymal phenotypes [170]. The differences in attribute, expression pattern, regulation, target gene signature, and function among EMT-TFs may determine the differential hierarchical role of EMT-TFs in cancer biology [43]. In addition, the EMT-TFs have the ability to modulate each other in a complex, dynamic and interdependent manner. The ablation of one EMT driver could be compensated by an alternative EMT program [187]. Investigating the specific EMT-TFs activation in a given tumor type is critical to defining the precise role of EMT in tumor progression and metastasis.

### The role of partial EMT

As a continuous flux between the extreme states of epithelial and mesenchymal, the process of EMT could be transited and reversible [43]. The binary transition between epithelial and mesenchymal phenotypes has been considered as the basis for EMT and MET. Recent studies have demonstrated that EMT is not a linear process but a spectrum and cancer cells are usually in a state between epithelium and mesenchyme [6, 14, 192]. Based on the spectral characteristics of EMT, related studies have successively introduced scoring algorithms for EMT. One study quantitatively estimated and scored the degree of EMT of tumor cell lines by the generic characteristics of EMT and correlated it with the efficacy of patient survival and drug response to assess patient prognosis [193]. Another study scored the partial EMT gene expression indexes based on genomics and proteomics, characterized and quantified the EMT state of the patient's tumor cells, and analyzed the survival of the patients [194].

**Fig. 4:**
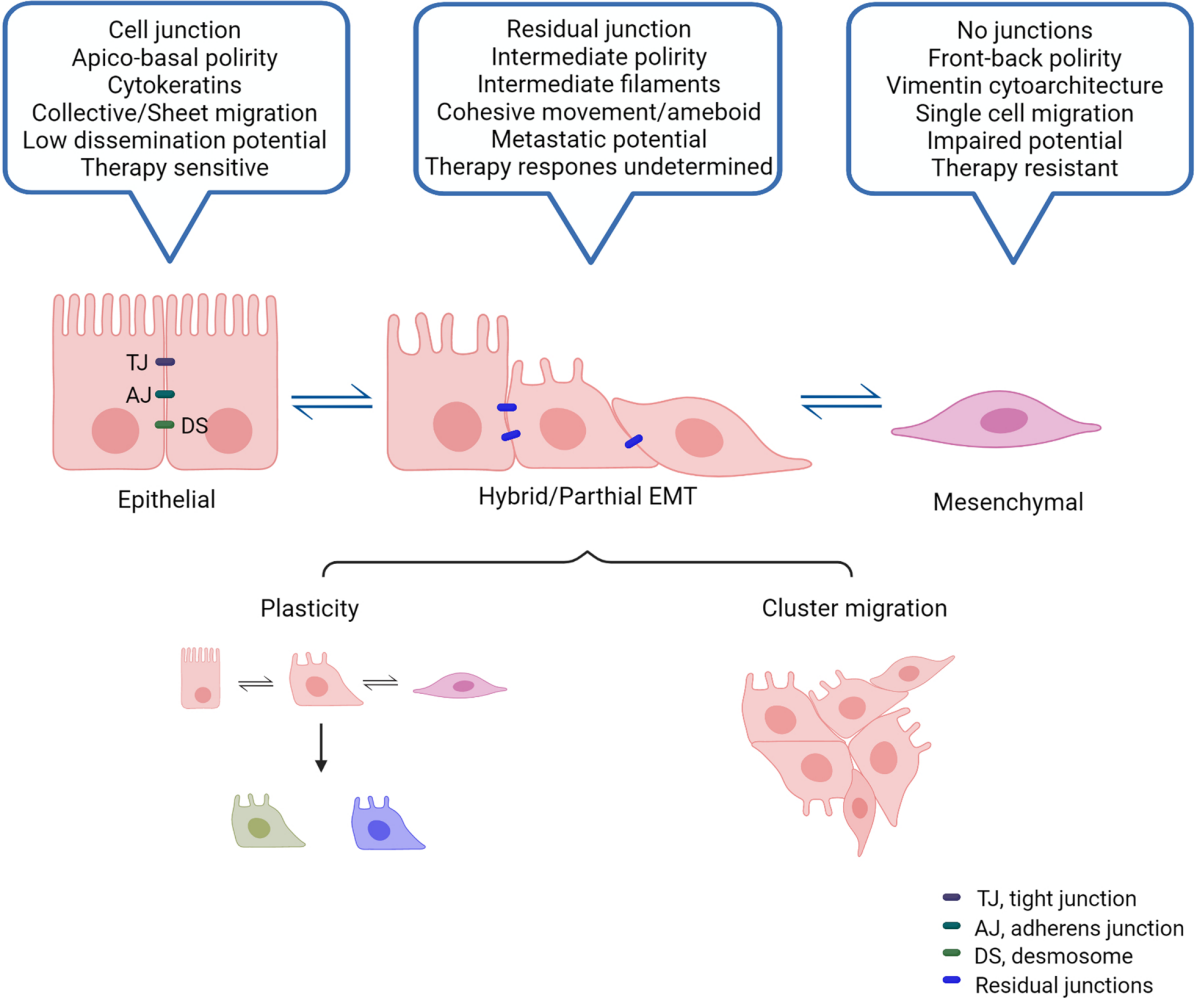
Features of partial EMT. The partial EMT as an intermediate state is not only phenotypically different from the epithelial and mesenchymal states, but also has greatly altered cellular properties. There is a synthesis of the literature; it has intermediate polarity and loose intercellular junctions compared to the two and has metastatic potential, but the response to treatment is not clear. In addition, partial EMT has plasticity and can differentiate into different cells and move in clusters within the body

Epithelial and mesenchymal markers are co-expressed or epithelial markers are absent without acquiring mesenchymal markers as a common manifestation of partial EMT, and this can place cancer cells in a dynamic window that may endow them with higher epithelial–mesenchymal plasticity for tumor progression and metastasis [43]. A recent study using a genealogical labeling approach and sequencing found that CDH1 epithelial protein was internalized in most tumors in the RAB11+ cycle, whereas at the protein level CDH1 mRNA remained unchanged in most cells [195]. Additionally, it has been found that tumor cell lines with partial EMT can produce clusters of circulating tumors in vivo and collectively migrate in vitro, whereas tumor cell lines with full EMT spread in form of single cells both in vivo and in vitro. In the KPCY model, it was found that cells in complete EMT often invaded and spread as single cells, while cells in the partial EMT stage often migrated more aggressively as a collective [195]. In the MMTV-PyMT mouse model of primary tumors, tumor cells rarely undergo a complete EMT program. Instead, they undergo partial EMT and can migrate, invade surrounding tissues at high rates, initiate metastatic growth, and return to an epithelial state to localize to distal organs via the MET program [196]. The application of the single-RNA sequencing technique can help us to delineate a clearer picture of EMT programs in tumor progression. Through single-cell sequencing technology, it was found that partial EMTs under-express SNAIL1/2, ZEB1/2, and TWIST1/2 during mouse organogenesis which is consistent with epithelial gene expression and they may be a transient population during development [197]. Puram et al. identified a subset of malignant cells with a gene signature associated with partial EMT in primary and metastatic head and neck squamous cell carcinomas using the single-RNA sequencing technique [198]. Interestingly, although these partial EMT cells exhibited several classical EMT features such as the expression of VIM, TGFβ-induced (TGFBI), and extracellular matrix genes, the EMT-TFs expression was significantly low, and the expression of the epithelial gene was still maintained [198]. Therefore, this confirmed the aggressive and highly plastic characteristics of partial EMT cells [198]. In a study conducted by Pastushenko et al*.* different combinations of the markers CD106, CD51, and CD61 were used to identify different states of tumor transition that occur during the EMT of cancer progression, distributed throughout the EMT spectrum, and introduction of the term “hybrid” EMT [14] (Fig. 4). Computational modeling studies have also revealed nonlinear multistable EMT dynamics. Besides, it was indicated that the intermediate hybrid EMT state is regulated by the feedback loops at the core of the EMT regulatory network, particularly the mutual inhibitory loops between several miRNAs and EMT-TFs [6, 199–204]. Future studies should focus on the dynamics of EMT spectrum, which will further clarify the role of EMT program in tumor metastasis.

## EMT and circulating tumor cells

Circulating tumor cells (CTCs) are cancer cells that have spread from the primary tumor to various organs through the vascular or lymphatic system [205]. Many studies have confirmed that certain CTC subpopulations of EMT metastases contain prognostic information. Circulating tumor cells represent a heterogeneous group, and the various hybrid phenotypes of EMT from the epithelium (E) to mesenchyme (M) play significant roles in tumor heterogeneity [13, 199]. Therefore, many EMT-related target genes can be used as the key indicators for CTC detection, among which vimentin and several epithelial adhesion molecules (E-cadherin, N-cadherin, and EpCAM among others) are frequently analyzed in CTC [167, 206, 207]. The ZEB (ZEB1 and ZEB2), SNAIL (SNAIL and Slug) family, and the TWIST family which are the core transcription factors regulating EMT target genes, are also frequently used as indicators for CTC assessment [6, 43, 208].

Because of the dynamic and reversible nature of EMT, it can improve the restrictive environment for tumor cell metastasis and EMT also affects the metastatic ability of the CTCs. First, EMT can promote CTC release; on the one hand, EMT stimulates angiogenesis and hence it has also been found that powerful angiogenic factor VEGF-A, which is an EMT target gene, is expressed in CTCs from patients with breast cancer [209]. In addition, EMT can promote the migration of tumor by inducing protein hydrolases such as matrix metalloproteinases (MMPs) [210]. High MMP activity has been reported in CTCs isolated from patients with prostate cancer [211]. A study found that platelets and EMT process jointly regulate intravasation/extravasation of CTCs [212]. During the process of intravasation, platelets bind to CTCs to release related cytokines, which promote the separation of tumor cells from the primary site and infiltrate into the blood and enter the circulation. Platelet-derived TGF-β and platelet-derived growth factors can drive the EMT process, thus endowing CTCs with metastasis and survival. The invasive ability enables CTCs to penetrate the vascular barrier to achieve extravasation.

The CTCs undergoing EMT are better able to resist apoptosis or anoikis because the EMT activates many survival pathways [213]. Typical EMT markers such as EMT transcription factors or stem cell markers which are related to molecules of survival pathways (such as EGFR, Akt, PI3K Bcl-2, and p53) were detected in CTCs isolated from colorectal, ovarian, breast, and other cancers [214–217]. Besides, clusters of CTCs that enter the circulation by the way of EMT may have different cellular phenotypes. A different study found that among the isolated CTCs, there were both cells with CTC clusters, cells with mesenchymal markers (including fibronectin, N-calmodulin, or PAI-1), and epithelial cell markers (such as EpCAM or cytokeratin), and the most of CTCs are EpCAM positive types [170].

Hordes of emergent EMT hybrid CTCs may establish the ability of cell–cell interactions to contribute to better resistance to anoikis/apoptosis and pro-transfer EMT-driven properties [218]. Therefore, CTCs travel in the form of a cluster with hybrid phenotypes of EMT may exhibit better survival and higher seeding efficiency in bloodstream and secondary sites. By expressing tissue factor (TF), the CTCs undergoing EMT will also be more effective in activating coagulation and building a protective cocoon. Tissue factor (TF) can be a target gene for EMTs and the EMT core transcription factors ZEB1 as well as SNAIL can regulate TF expression, with vimentin stabilizing TF mRNA [219, 220]. The TF/EMT relationship has also been confirmed through the correlation between vimentin and TF expression in studies of triple-negative breast cancer (TNBC).

Furthermore, the EMT induces multiple receptors that mediate the interaction of neutrophils with CTCs or CTCs with platelets/fibronectin (including CD44, ICAM1, αvβ3, or VCAM1), thereby driving cell cycle progression in the circulation system and expanding the metastatic potential of CTC [221–226]. Neutrophils have also been shown to induce EMT in several cell systems through the release of soluble factors, including CXCL-1, IL-17, or neutrophil elastase [227–229]. In addition to the above mechanisms, EMT can also induce immune escape, including increased expression of immune checkpoint proteins, altered autophagy, immunoproteasome defects, and immune synaptic dysfunction [230, 231].

A large number of clinical studies such as those in breast, prostate, and rectal cancer support the use of CTC counts as a valid prognostic biomarker before or during cancer treatment (chemotherapy or targeted therapy) [232–236]. Previous studies have shown that certain CTC subpopulations of EMT metastases have prognostic information and hence prognostic-related information can be found by detecting other typical EMT markers [237, 238]. A correlation between PD-L1 expression and EMT markers has also been demonstrated in tumors and CTCs, particularly in NSCLC and TNBC [239]. Therefore, combined detection of known therapeutic targets such as EGFR, PD-L1, or HER2 as well as EMT markers with poorer prognosis could point to potential combination therapies and improve patient care [240]. Furthermore, the emergence of CTCs phenotypes undergoing EMT correlates with drug resistance and the use of EMT as a concomitant marker in the treatment process may help predict the development of resistance and guide clinical treatment strategies [241–243]. There is still a discussion about EMT signatures in CTCs. Therefore, the selection of appropriate CTC isolation techniques, specific EMT markers, and transfer to determine clear parameters for clinical routine are important for future clinical translational application as well as the targeted therapy of CTC for EMT.

## Relationship of EMT and tumor stemness

To explain how the propagation and dissemination of completely heterogeneous tumors at secondary sites is achieved by CSCs, the induction of EMT and stemness at the front of tumor invasion was firstly proposed [244]. Many epithelial tissues are maintained by stem cells that exhibit two remarkable phenotypes: one is the ability to differentiate and generate daughter cells with specific functions associated with epithelial phenotypes, whereas the other is the ability to self-renew, thus maintaining the stem cell pool [245]. In this section, we discussed the relationship between EMT and tumor cell stemness.

The CD44, CD24, CD133, and aldehyde dehydrogenase 1 (ALDH1) are the commonly used CSC surface markers. In the breast, EMT-derived stem cells are phenotypically similar to CSCs after induction, expressing CD44^High^, CD24^low^, and forming mammospheres [221]. Since then, this feature has been found in many CSC subpopulations and other studies have reported that high intracellular ALDH1 activity is a marker of stemness [246, 247]. Recent studies have shown that the activity of CD44^high^ and ALDH1 do not usually coexist in CSC, which reveals the presence of different types of CSC [248]. A separate previous study focused on lung cancer with tobacco carcinogens exposure found a remarkable phenomenon. It was evident that CD44^high^/ CD24^low^ cell population acquired a dramatic increase under brief carcinogen exposure. Further, the expression of CD133 and ALDH1 also improved but not significantly [249]. However, the expression of stem cell markers is not always consistent in the same subtype of breast cancer [250]. Therefore, the combination of CD44 subtype binding ALDH1 activity and integrins is promising in the study of primary tumors and their metastatic proliferation.

In addition, the EMT process plays a crucial role in enriching the CSCs pool in breast cancer. Specifically, PGC-1α and miR-200c are progressively inhibited by the EMT process and this results in mitofusin 1 aggregation. The NUMB gains phosphorylated and then dissociates from the cortical membrane with mitochondrial fusion, and the stem cells undergo asymmetric division to ensure a sustained CSC pool [251].

### Signals of EMT-induced stemness

CSC are tumor cells with key characteristics of self-renewal, tumor initiation potential, and clonal long-term repopulation potential and are a small subset of malignant cells in tumors [252]. Further, the acquisition of stem cell properties is associated with the activation of EMT. Several EMT-TFs are involved in the stemness regulation of tumor stem cells as interference signals and are key regulators of CSC [253].

Among the different EMT-TFs, ZEB1 was the first one that was investigated in pancreatic cancer as a link from EMT to stemness. The experiment found that lowering ZEB1 causes pancreatic cell lines to lose the ability to form tumor spheres in vitro, and tumor initiation in vivo, hence confirming the requirement of stemness for Zeb1 [254].

Bmi1 is an epigenetic regulator that plays an important role in the maintenance of adult stem cell function and can also induce EMT [255]. Generally, Bmi1 is modulated using miR-200/-205 by binding 3’UTR sequence. Therefore, miRNA families repress CSC stemness through Bmi1 suppression-resulted cell apoptosis, senescence, and differentiation [256, 257]. Bmi1 can also be indirectly repressed by ZEB1. Furthermore, previous studies have shown that ZEB1 directly represses the miR-200 family, which in turn represses the expression of Bmi1 [258, 259]. The results explain how EMT regulates stem cells in this context and reveal a ZEB1/miR-200/Bmi1 pathway in pancreatic CSCs.

Upon inhibition by H3K27me3, the promoter of miR-200/-205 gets methylation, which relieves ZEB1 and increases EMT [249]. In addition, Bmi1 binds to PTEN (phosphatase and tensin homolog) at human nasopharyngeal epithelial cells and initiates PI3K/Akt/GSK-3β signaling to promote EMT in the nasopharyngeal carcinomas process [256]. Elsewhere, the pathway by which the ZEB1/miR-200/Notch signaling axis regulates CSCs was also elucidated [260].

The ability of pancreatic cancer cells to form tumor spheres was reduced through inhibition of the Notch pathway (achieved using γ-secretase inhibitors or knockdown of Jag1/Maml2/Maml3) suggesting that cell stemness of CSCs can be induced through the Notch pathway [261]. Notch signaling components Jag1, Maml2, and Maml3 are direct targets of miR-200 inhibition in breast and pancreatic cancer cell lines. Moreover, it has been found that Zeb1 inhibits miR-200 family members, resulting in de-repression of the expression of the three signaling components [262].

In the context of head and neck squamous cell carcinoma (HNSCC), it was found that EMT-TF, Twist can control CSC stemness by signaling to Bmi1. It directly binds the Bmi1 promoter and activates Bmi1, and hence TWIST1 can upregulate BMI1. Both the tumor spheroid and tumor initiation properties of HNSCC cells can be induced by Twist and Bmi1, and thus Bmi1 is essential for the stem induction ability of TWIST in these cells [81]. In epithelial ovarian cancer (EOC), TWIST1 is regulating the miRNAs miR-199a and miR-214 to control stem cell differentiation, and these miRNAs can control cell proliferation, apoptosis, and inflammation [263].

Of the SNAIL family, EMT-TF is not only associated with the stemness characteristics of the multilayered epithelium, but may also be involved in its signaling regulation [264]. It was found that the ability of SNAIL to significantly induce tumor spheres and tumor initiation in rectal cancer cell lines was the highest expressed EMT-TF. Expression of Numb which is an inhibitor of the Wnt signaling effector protein β-catenin can be inhibited by miR-146a. Further, it was evident that the SNAIL bound and activated the promoter of miR-146a, which in turn promoted the activation of the Wnt pathway, fully validating it as a stem regulator. Therefore, they did not only confirm the role of the SNAIL/miR-146/Numb/β-catenin pathway in promoting SC properties, but also found a poor prognostic and treatment resistance correlation of SNAIL High NUMB Low in a group of patients with colorectal cancer in a clinical study [265]. Elsewhere, it has been found that Slug which is a member of the SNAIL family, can induce mammary stem cell (MASC) through synergistic activation of different autoregulatory gene expression programs with sox9 [266]. Therefore, stemness and mesenchymal properties of tumor cells were found to be maintained through the KLF4/TGF-β1/Smad/SNAIL pathway in a human colorectal cancer model [267].

Although many reports suggest that cells undergoing full EMT may acquire the stemness, the relationship between complete EMT coupled with stemness has been challenged by some studies [268–272]. To reconcile these conflicting results, existing studies suggest that cells in a mixed E/M or partial EMT state are more likely to acquire stemness than cells in a pure epithelial or mesenchymal state [273–276]. This model is supported by several lines of evidence from studies of sphere formation and tumorigenicity in prostate and breast cancer models [6, 221, 268, 274]. Furthermore, in an ovarian cancer model, it has been found that the partial EMT phenotype increased tumor stemness, whereas loss of stem cell markers and tumorigenicity was associated with a fully epithelial or fully mesenchymal phenotype [275]. This implies that there is a “stemness window” between fully differentiated epithelial cells and fully differentiated mesenchymal cells [199, 277]. In other words, cells with a partial/hybrid EMT phenotype, rather than cells locked in a full EMT phenotype, have higher plasticity in tumor invasion and proliferation and can complete the invasive metastatic cascade.

In a clinical study, the subpopulations of CTC were analyzed according to stem and EMT markers after chemotherapy. It was found that only CSC^+^/partial EMT^+^ CTCs (co-expressing stem and partial EMT phenotypes) were highly enriched after chemotherapy which was associated with pulmonary metastases and lack of treatment response. Therefore, CSC^+^/partial EMT^+^ CTCs can be used as a prognostic marker for metastatic breast cancer patients receiving first-line chemotherapy [242]. In addition, effectively targeting a subpopulation of CTCs with stem cell properties and high metastatic potential has the potential to improve patient survival and may be a promising avenue for cancer treatment.

## Therapeutic strategies and challenges for EMT

The EMT may be resistant to a variety of treatments, including chemotherapy, radiotherapy, and activation of EMT controls resistance to treatment at multiple levels [278]. Increased drug efflux or avoidance of apoptosis and necrosis are the common pathways of drug resistance [19]. As an important example, SLUG and SNAIL can avoid treatment-induced apoptosis by interfering with p53 function or inhibiting the tumor suppressor PTEN (phosphatase and tensin homolog) [213]. Two separate studies have found that in genetically engineered mouse models of breast and pancreatic cancer, the primary and metastatic tumor cells became resistant to chemotherapeutic agents with an EMT-dependent presentation [185, 186]. Further, the activation of EMT also confers carcinoma cell ability to induce local immunosuppression, hence compromising immunosurveillance and contributing resistance to immunotherapy. Mesenchymal carcinoma expressing EMT markers exert immunosuppressive effects in multiple ways, including secreting chemokines and cytokines, thus promoting the formation of regulatory T (Treg) cells, recruiting M2 macrophages, blocking cytotoxic activities of T lymphocytes (CTL) and natural killer (NK) cells, and inhibiting antigen presentation of dendritic cells (DCs) [110, 279–282].

Notably, EMT in carcinoma cells promotes the expression of PD-L1 and a higher EMT score correlates with tumors that respond best to CTL-A4, PD1, and PD-L1 antibodies and with tumors that express other increased immune checkpoint markers [193, 283–285]. In addition to inducing resistance to various treatments, EMT can also be induced after treatment, including activation of EMT-promoting pathways via TGF-b, NF-κB, WNT, FGF, and EGF/HER2. Therefore, induction of EMT may exhibit adaptations in response to treatment-induced cellular stress [230, 286]. Further, the activation of EMT after treatment leads to further acceleration of the disease process through mechanisms including increased proliferation, decreased apoptosis, immunosuppression, increased stemness, and metastasis [286].

**Fig. 5:**
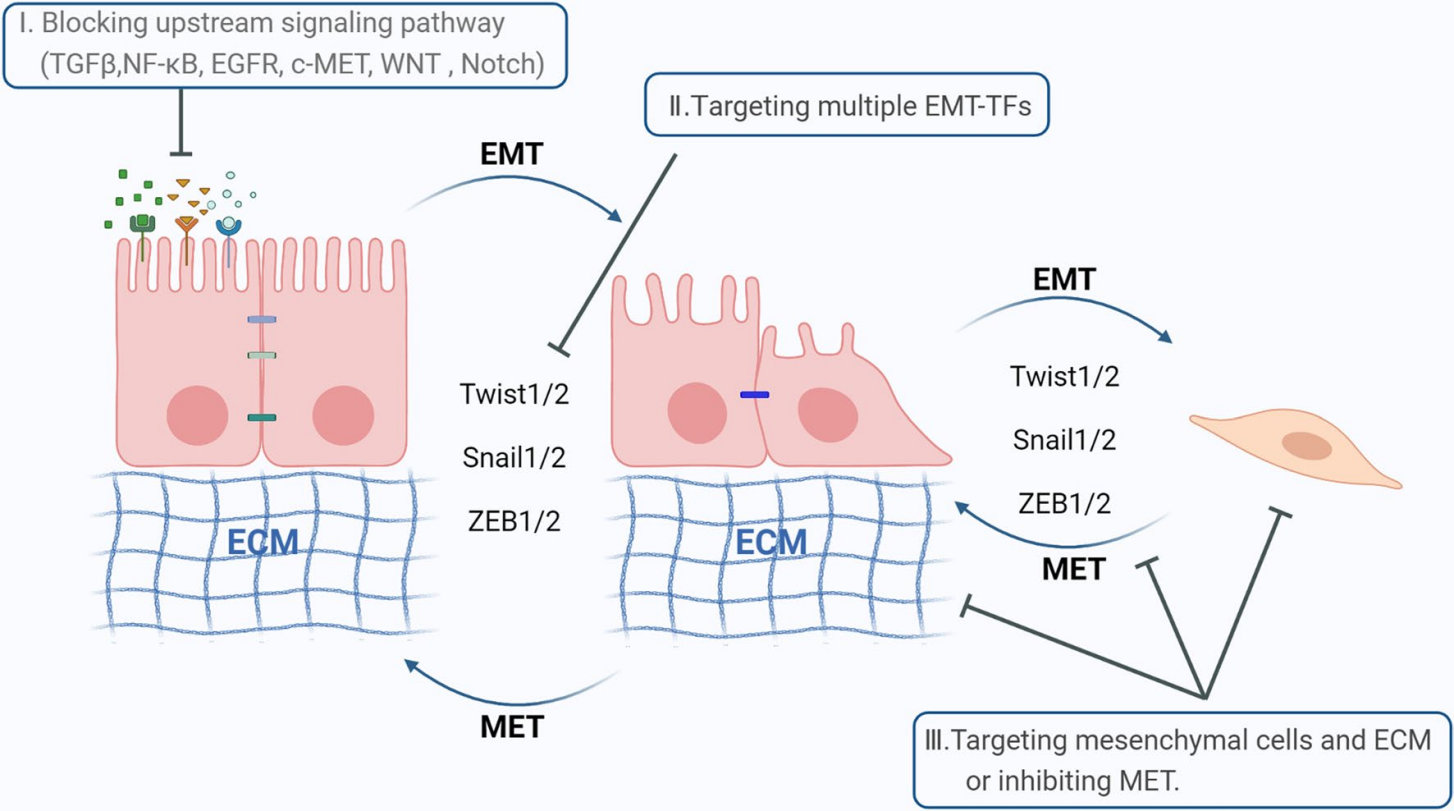
Therapeutic strategies for targeting EMT. EMT may be resistant to various treatments such as chemotherapy and radiotherapy, and the activation of EMT after treatment leads to further acceleration of the disease process by mechanisms including increased proliferation, decreased apoptosis, immunosuppression, stemness, and increased metastasis. There are three main strategies for targeted EMT treatment: I) inhibiting tumorigenesis by blocking upstream signaling pathways, II) targeting the molecular drivers of EMT, and III) targeting mesenchymal cells and outer stroma or inhibiting the MET process

Prevention or reversing the lethal effects of EMT is of great importance for cancer treatment. Currently, there are three main strategies for targeted EMT therapy (Fig. 5) (Table 1). First, it can inhibit tumorigenesis by blocking upstream signaling pathways. This includes ligand-neutralizing antibodies, decoy receptors, or inhibitors blocking TGFβ, NF-κB, EGFR, cMET, WNT, and Notch signaling [286–288]. In addition, effective inducers of EMT include a variety of pro-inflammatory signals such as TNF-α [289]. Another therapeutic strategy is to target the molecular drivers of EMT. Although EMT- TFs are the main drivers/regulators of the EMT process, direct targeting of the transcription factors (EMT-TFs) is challenging [68]. Further, several EMT-TFs have a complementary and redundant function because they tightly connect via feedback mechanisms. Therefore, targeting their interactions with important cofactors may be a more beneficial strategy while also targeting multiple EMT-TFs [68].

Targeted regulation of epigenetics is a reversible and stable way of inducing the reprogramming required for phenotypic switching in the EMT process [290]. The application of modified synthetic miRNAs that can interfere with EMT-TF at the post-transcriptional level, such as liposomal miR-34 (MRX34) on tumor growth and metastasis has been previously used in clinical trials [291].

Another therapeutic strategy is to target mesenchymal cells and ECM or inhibition of the MET. Inducing re-differentiation or trans-differentiation by inhibiting the function of mesenchymal-specific proteins, blocking cell plasticity, and re-sensitizing tumor cells to standard treatments. For instance, using biologically active compounds or monoclonal antibodies against vimentin, fibronectin, and N-cadherin [292]. A study has found that there is a group of mixed epithelial/mesenchymal phenotypic cells called quasi-mesenchymal (qM) cells in the EMT process, which will metastasize and become resistant to immune checkpoint blocking therapy. In this study, knockout-derived factors (CD73, CSF1, or SPP1) targeting qM cancer cells can prevent the assembly of immunosuppressive tumor microenvironment, promote their transition into epithelial phenotypic, and improve the sensitivity to checkpoint immunotherapy [293].

Another therapeutic strategy is to target the interaction between cancer cells and ECM. Integrins, a family of ubiquitous cell membrane adhesion receptors, play an imperative role in physiological processes via their attachment to the ECM [294]. The interaction between cancer cells and the ECM are mostly mediated by integrins, which further activates the signals involved in the initiation, progression, and metastasis of solid tumor [288, 294]. Thus, integrins present themselves as an attractive target for cancer treatment due to their crucial role in tumor progress and metastasis. Indeed, inhibiting the function of integrin was shown to reduce tumor metastasis in animal models and maintain a stable disease state in clinical trials. [288, 294–298]. In parallel with the launch of EMT program in cancer cells, the degradation of ECM is initialed by the synthesis of proteases of the MMPs family, which further allows cancer cells to enter the circulatory system and implant in distant tissue [299]. In summary, several potential anti-cancer drugs MMPs and their inhibitors have been extensively studied [300, 301].

However, research on targeted EMT treatment strategies is challenging and the serious adverse effects caused by EMT-targeted therapy cannot be neglected. Targeted EMT therapy can inhibit the migration and invasive behavior of tumor cells in the primary tumor, but this approach only works if the early spread of cancer cells has not occurred [302, 303]. In two studies of genetically engineered mouse models of breast and pancreatic cancer, primary and metastatic tumor cells, EMT was shown to confer stronger resistance to chemotherapeutic drugs [185, 186]. Therefore, in the early stage of cancer development, EMT-targeted therapy combined with conventional chemotherapy can improve the sensitivity of tumor cells to drugs. EMT-targeted therapy can also prevent tumor cells from further spreading, and lock them in the primary site with a clear boundary, which is beneficial to the resection of the primary tumor. In the later stages of the disease, EMT inhibitors can reduce the proportion of CSCs in tumors, prevent CTC colonization of the primary tumor and reduce the ability of CTCs to generate secondary tumors when seeded at distant sites [278, 304]. In a study of triple-negative breast cancer, a small molecule inhibitor of EMT, GSK-3β inhibitor BIO, could effectively and selectively inhibit the EMT and CSC, and migration characteristics of cells with mesenchymal and stem cell phenotypes [305]. When tumor cells lose their CSC properties, the expression of ABC proteins will decrease, reducing drug efflux and increasing the efficacy of chemotherapy [288]. It is still not clear whether patients, with early-stage disease or with advanced disease (with CTC detection and evidence of distant metastases) will benefit most from anti-EMT therapy. Therefore, this makes the selection of the appropriate timing of targeted EMT to be critical. Anti-EMT treatment may induce a contrary result than originally expected. The reverse process may be driven during anti-EMT treatment, leading to the development of MET, promoting the colonization, and metastasis of circulating tumor cells, and increasing tumor proliferation will make cancer cells more susceptible to chemotherapeutic agents [32]. As EMT plays very imperative physiological roles, the targeting process of EMT not only has an effect on tumor cell subpopulations but may also have a negative impact on normal cells [24]. In addition, inhibition of the EMT process may affect the repair function of the body because EMT plays an important role in the physiological response to trauma and wound healing [164]. Therefore, the targeted therapy of EMT has two sides and the research process is concerned about its effect, whereas the consequences of side effects are also worthy of attention.

## Conclusion and perspectives

EMT is a highly regulated dynamic process that is angelic and demonic to an organism. During embryonic development and tissue repair, EMT is an essential presence, but the promotion of this process by EMT during tumor development often has undesirable consequences [18, 42]. It confers motility, stem cell properties, and therapeutic resistance to epithelial cells in a variety of normal and cancerous tissues. This process can be activated by a variety of signals and a regulatory network of multiple transcription factors and processes such as post-transcriptional and posttranslational modifications as well as epigenetic modifications govern the execution of EMT.

Regardless of our growing familiarity with the EMT program, the precise requirement of EMT and EMT-TFs in tumor metastasis is still debatable [185–189]. These contradictory results collectively demonstrated that the different attributes and expression patterns in different tumor types determine their precise role and function in cancer biology. A clear illustration that EMT effectors are in diverse cancer types should be further delineated. It is also worth noting that EMT in tumors is a spectrum of intermediate states rather than a binary process. Tumor cells often show a partial EMT phenotype that exhibits various degrees of epithelial and mesenchymal markers expressions [187]. Thus, it might be defective to fully capture all the ongoing EMT events (such as partial EMT) by tracing cells based on the single gene expression as described in previous studies [185–187]. Future studies should combine some omics approaches such as the single-cell RNA sequencing technique to dissect the dynamics and involvement of partial EMT precisely and comprehensively in tumor metastasis.

CSCs in the EMT process place tumor cells in an intermediate state of the E to M spectrum, a state in which cells are highly resistant to chemotherapy and can survive and generate new tumor cells that eventually leads to clinical relapse [17, 306]. In addition, increased drug efflux and avoidance of apoptotic signaling pathways have also been suggested as relevant resistance mechanisms for EMT [307]. EMT contributes to immunosuppression within the immune microenvironment, and activation of EMT-TFs leads to the accumulation of immunosuppressive cells in the tumor microenvironment. For instance, a related study found that activation of SNAIL in ovarian cancer upregulates CXCL-1 and CXCL-2, and activation of ZEB1 in breast cancer upregulates IL-6 and IL-8, both of which recruit MDSCs to the tumor microenvironment, leading to immunotherapy resistance and promoting cancer progression [308, 309]. Moreover, immune cells can secrete cytokines and chemokines to regulate EMT, and tumor cells that receive EMT can in turn produce immunosuppressive cytokines or chemokines. These two processes can complement each other to further promote cancer progression [310]. Therefore, immunotherapy targeting immunosuppressive cells in combination with immune checkpoint inhibitors represents a promising anti-cancer therapy .

**Table 1:** Inhibitors of EMT in clinical phase trials

Treatment strategies	Target	Drug	Stage/phase	Cancer	Trial identifier
I. Blocking upstream signaling pathway	TGF-βR1	Galunisertib monohydrate	II	(Metastatic) breast cancer	NCT02538471
	TGF-βR1	Vactosertib	I	Bladder cancer, breast cancer, melanoma, and prostate cancer	NCT03704675
	TGF-βR2	SHR-1701	I	Solid cancers	NCT04324814
	NF-κB	Bardoxolone methyl	II	Melanoma	NCT00535314
	EGFR	Gefitinib	Marketed	Breast cancer and NSCLC	
	EGFR	Panitumumab	Marketed	Metastatic colorectal cancer	
	c-MET	Glesatinib	III	Non-small cell lung cancer	GDCT0241492
	c-MET	Cabozantinib s-malate	Marketed	Medullary thyroid cancer	
	WIF1	BI-1361849	I and II	Non-small cell lung cancer	GDCT0251874
	WIF1	OXB-301	III	Renal cell cancer	GDCT0017470
	NOTCH2	Tarextumab	I/II	Pancreatic cancer Small cell lung cancer	NCT01647828 NCT01859741
II.Targeting multiple EMT-TFs	HDAC1/4	Mocetinostat	II/III	Bladder cancer	NCT02236195
	HDAC1/2/3/6	Vorinostat	III	Lung cancer	NCT00419367
	LSD1	Domatinostat	II	Small cell lung cancer	GDCT0245900
	LSD1	INCB-59872	I	Ewing sarcoma	NCT03514407
	ZEB1/SNAIL2/TWIST/Vimentin	Metformin	II	Pancreatic	
III. Targeting mesenchymal cells and ECM or inhibiting MET	N-cadherin	ADH-1	II	Adrenocortical carcinoma and non-small cell lung cancer	NCT00264433
	Fibronectin	Monoclonal antibody	II	Breast, colorectal, lung and non-small cell lung cancer	NCT01125085
	Vimentin	Pritumumab	II	Brain cancer	GDCT0217875
	MMP2/3/9/13	Tanomastat	III	(Non)-small cell lung cancer	GDCT0022508 GDCT0023988
	MMP	Batimastat	I	Hepatocellular	
	FAK	APG-2449	I	Non-small cell lung, ovarian cancer	GDCT0352706
	EpCAM	Catumaxomab	I	Epithelial carcinomas	
	ITGA5/B1	ATN-161	II	Renal cell cancer	NCT00131651
	ITGA5/B1	Volociximab	II	Renal cell cancer	NCT00100685
	ITGAV	Cilengitide	II	Glioblastoma multiforme	NCT01782976
	ITGAV	Intetumumab	II	Prostate cancer	NCT00537381

Although the involvement of EMT in invasion, dissemination, and extravasation is essential for the propagation of primary tumors, EMT alone cannot complete the metastatic colonization of distal organs using tumor cells but requires the subsequent process of MET to restore the epithelial phenotype to complete the growth of metastatic lesions [14, 311]. Therefore, EMT-TF is variable and tissue-specific in promoting metastasis as well as essential in some cases, but not necessary in others. However, this evidence refutes the view that EMT is non-essential for tumor metastasis. The SNAIL has been shown to have such properties in breast cancer and TWIST1 in squamous cell carcinoma [182, 271]. The dispensability of EMT is dependent on the dominant role of EMT-TF and there may be a hierarchical relationship between EMT-TF. In addition, different tumors have very different effects on EMT-TF and EMT, and it would be crucial to evaluate the step of EMT works in which tumor, which would be the key to blocking EMT or clearing EMT.

Neither is EMT a single-cell state nor a binary process but a mixture of different types of cell states, which provides a “stem cell window” and makes EMT plastic, especially partial EMT [312]. Related studies have shown that different EMTs have different characteristics of infiltration, metastasis, and differentiation, when the cells of mixed epithelium and mesenchyme play a greater role in reaching circulation, colonization, and metastasis [14]. The mixtures of epithelial and mesenchymal subpopulations can be distinguished from exclusively hybrid E/M cells based on gene expression [313]. Subpopulations of EMT are spatially distributed in specific tissues, non-randomly in various parts of the tumor, exist in different microenvironments, and are associated with different stromal cells. It is now widely believed that some of the cells produced by partial EMTs contain mixed epithelium and mesenchyme which is a key factor in the invasion and spread of cancer cells. Further, this promiscuous feature also plays a crucial role in the successful metastatic colonization of disseminated cells [33]. Therefore, deciphering the full morphology of EMT and the transformation of cells between different states will help in understanding tumor heterogeneity, growth, invasion, metastasis, and drug resistance.

For cancer, although conventionally targeted approaches can be used or the tumor microenvironment can be used for treatment, plasticity of EMT can also lead to drug resistance. Therefore, it is a great challenge to target and destroy the cells that receive EMT in tumor tissue. Inhibition of EMT may inadvertently promote secondary tumor formation because of the plasticity of the procedure and the need for tumor cells to implant in distant organs requiring a return to an epithelial state. Alternatively, a trans-differentiation strategy can be used to induce EMT cells into a harmless cell and push them to an extreme EMT state, leading to their eventual differentiation or apoptosis and this has also been shown to be effective in breast cancer-related studies [312]. In a breast cancer model, it was observed that the infiltrating breast cancer cells can give rise to adipocytes by trans-differentiation, thereby inhibiting cancer metastasis. Moreover, targeting downstream effectors of EMT/MET is a more accurate therapy. During MET, the miR-200s downregulates Tinagl1 (a secreted metastasis inhibitory protein) and thus recombinant Tinagl1 reduces tumorigenesis and metastasis, whereas EMT induction is prevented by directly targeting miR-200 s [314]. Whatever, researchers should continually investigate the role of EMT in tumor development, and develop better treatments for targeting the EMT process.

## Data Availability

Not applicable.

## Abbreviations

EMT: Epithelial–mesenchymal transition
MET: Mesenchymal–epithelial transformation
TFs: Translational factors
BHLH: Basic helix–loop–helix
CSCs: Cancer stem cells
CTCs: Circulating tumor cells
PAR: Partitioning‑defective
PATJ: PALS1-associated tight junction
ZO1: Zonula occludens 1
MMPs: Matrix metalloproteinase
PRC: Polycomb repressor complex
EZH2: Enhancer of zeste homolog 2
LSD1: Lys-specific demethylase 1
RTKs: Receptor tyrosine kinases
CTBP: C-terminal-binding protein
SWI/SNF: Switch/sucrose nonfermentable
TGF-β: Transforming growth factor-β
BMP: Bone morphogenetic protein
BMI1: B lymphoma Mo‑MLV insertion region 1 homolog
HIF-1α: Hypoxia-inducible factor-1α
FOX: Forkhead box
SOX: SRY box
PRRX1: Paired-related homeobox 1
YB-1: Y-box-binding protein-1
CPEB1: Cytoplasmic polyadenylation element-binding protein 1
GSK-3β: Glycogen synthase kinase-3β
PKD1: Protein kinase D1
SCP1: Small C-terminal domain phosphatase 1
PAK1: P21-activated kinase 1
LATS2: Large tumor suppressor 2
USP51: Ubiquitin-specific protease-51
HMGA2: High-mobility group A2
3′-UTR: 3′ Untranslated regions
lncRNAs: Long noncoding RNAs
ET-20: EMT-associated transcript 20
MCSCs: Metastatic cancer stem cells
EMP: Epithelial–mesenchymal plasticity
uPA: Urokinase-type plasminogen activator
ICAM1: Intercellular adhesion molecules
TGFBI: Transformation of growth factor beta-induced
TNBC: Triple-negative breast cancer
ALDH1: Aldehyde dehydrogenase 1
HNSCC: Head and neck squamous cell carcinoma
EOC: Epithelial ovarian cancer
MASC: Mammary stem cell
HF: Hair follicle
CTL: Cytotoxic activities of T lymphocytes
NK: Natural killer
DCs: Dendritic cells
qM: Quasi-mesenchymal
